# Be_12_O_12_ Nano-cage as a Promising Catalyst for CO_2_ Hydrogenation

**DOI:** 10.1038/srep40562

**Published:** 2017-01-18

**Authors:** Haiyan Zhu, Yawei Li, Guizhi Zhu, Haibin Su, Siew Hwa Chan, Qiang Sun

**Affiliations:** 1 Singapore-Peking University Research Centre, Campus for Research Excellence & Technological Enterprise (CREATE), 138602, Singapore; 2 Institute of Modern Physics, Northwest University, Shaanxi Key Laboratory for Theoretical Physics Frontiers, Xi’an, 710069, China; 3 Department of Materials Science and Engineering, Peking University, Beijing, 100871, China; 4 School of Materials Science and Engineering, Nanyang Technological University, 639798, Singapore; 5 School of Mechanical and Aerospace Engineering, Nanyang Technological University, 639798, Singapore

## Abstract

An efficient conversion of CO_2_ into valuable fuels and chemicals has been hotly pursued recently. Here, for the first time, we have explored a series of M_12_x_12_ nano-cages (M = B, Al, Be, Mg; X = N, P, O) for catalysis of CO_2_ to HCOOH. Two steps are identified in the hydrogenation process, namely, H_2_ activation to 2H*, and then 2H* transfer to CO_2_ forming HCOOH, where the barriers of two H* transfer are lower than that of the H_2_ activation reaction. Among the studied cages, Be_12_O_12_ is found to have the lowest barrier in the whole reaction process, showing two kinds of reaction mechanisms for 2H* (simultaneous transfer and a step-wise transfer with a quite low barrier). Moreover, the H_2_ activation energy barrier can be further reduced by introducing Al, Ga, Li, and Na to B_12_N_12_ cage. This study would provide some new ideas for the design of efficient cluster catalysts for CO_2_ reduction.

The energy crisis and greenhouse effect caused by the emission of carbon dioxide (CO_2_) are the two serious global problems at the present day and even remain in the next 50 years^1^, which stimulated the current research interest in efficient conversion of CO_2_ into valuable fuels and chemicals^2^,^3^,^4^. However, due to the negative adiabatic electron affinity (EA) and large ionization potential (IP), the CO_2_ molecule is thermodynamically stable and kinetically inert, thus making the conversion difficult under normal conditions^5^. To overcome these challenges, we need to understand the basic chemical processes of the conversion and seek for highly efficient, cost-effective, and environmentally sound catalysts. Since formic acid (FA) has been widely used as a medium for hydrogen storage and an industrial chemical, catalytic hydrogenation of CO_2_ to FA becomes one of the most common and promising way to utilize CO_2_.

Recently, systems containing frustrated Lewis pairs (FLPs) have been found as effective catalysts for H_2_ activation^6^,^7^,^8^, CO_2_ reduction^9^,^10^,^11^ and hydrogenation^12^,^13^,^14^ for the production of C1 fuels. As we know that a FLP contains both Lewis acid and base centers, and the most common active Lewis pairs are B/N, B/P, Al/N and Al/P. Furthermore, a cationic Lewis acid component has also been extended to silicon^15^,^16^, carbon^17^, in ref. 9 and even the transition-metal (Zr^18^,^19^ and Ti^20^) complexes, while the Lewis base component has been extended to O^9^, carbenes^21^, ethers^22^, ketones^23^, and sulfides^24^. The reduction of CO_2_ via FLPs usually consists of two major steps: hydrogen activation and hydrogen transfer to CO_2_, where a hydrogen molecule is first split into a proton (H^+^) and a hydride (H^−^), and then CO_2_ is reduced via a concerted or sequential transfer of H^+^ and H^−^ to CO_2_. By theoretical calculations, Liu *et al*. found a relationship between these two steps, i.e. a stronger FLP results in a lower energy barrier for H_2_ activation, but in a higher energy barrier for H transfer^12^.

Inspired by the mechanism of CO_2_ hydrogenation by FLPs, here we raise a question: whether the clusters consisting of the active element for FLPs such as B/N, B/P, Al/N and Al/P etc. could act as catalysts for H_2_ splitting and CO_2_ further hydrogenation?

In the past few years, experiment and theoretical research efforts have been devoted to (XY) n (M = B, Al, Be, Mg; X = N, P, O) nanostructures such as nanocages, nanohorns, nanotubes, and nanowires^25^,^26^,^27^,^28^,^29^. Theoretical studies found that the fullerene-like cages (XY)_12_ with *Th* symmetry were the most stable geometry^30^,^31^. Moreover, B_12_N_12_ has been synthesized and detected by laser desorption time-of-flight mass spectrometry^32^. Al-, Ga- doped^33^ and Li-, Na- decorated^34^ stable B_12_N_12_ clusters have been also theoretically studied. In addition, previous studies indicated that BN^35^, AlN^36^,^37^ and BeO^38^,^39^ clusters can absorb H_2_ molecularly due to the polar bond between B and N, Al and N, Be and O with different electron affinities. Moreover BN clusters can also capture CO_2_^40^,^41^,^42^. In fact, the most special point for cluster catalysis is that the addition or removal of a single atom can have a substantial influence on the activity and selectivity of reaction, which provides us the basis for converting CO_2_ to different products with different efficiencies by introducing different atoms to the cluster surface or inside the cluster with more flexibilities and diversities. Based on these points, in the present work we systematically study H_2_ dissociation (2H*) and CO_2_ hydrogenation using M_12_x_12_ (M = B, Al, Be, Mg; X = N, P, O) cage clusters, and explore the involved mechanisms.

## Results and Discussions

### Geometry structures

**Figure 1:**
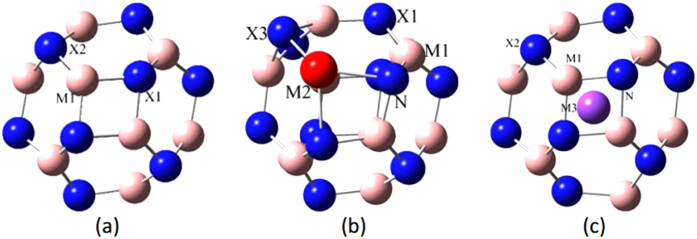
Geometry sketches. (**a**) MX clusters (M1 = B, Al, Be, Mg; X = N, P or O); (**b**) AlB_11_N_12_ and GaB_11_N_12_ (M2 = Al, Ga); (**c**) LiB_12_N_12_ and NaB_12_N_12_ (M3 = Li, Na).

For M_12_x_12_ type cluster with *T*_*h*_ symmetry as B_12_N_12_, Al_12_N_12_, B_12_P_12_, Al_12_P_12_, Be_12_O_12_ and Mg_12_O_12_, they all consist of eight 6-membered rings (6-MR) and six 4-membered rings (4-MR), as was shown on Fig. 1(a). To improve the binding with CO_2_ and H_2_, Al, Ga [Fig. 1(b)] and Li, Na [Fig. 1(c)] are introduced to B_12_N_12_ cage, the geometry parameters can be found in Table S1 of Supporting Information, where there are two types of M-X bonds: the bond shared by two 6-MRs (labelled MX-66), and the other one shared between a 4-MR and 6-MR (labelled MX-64), the former is longer than the latter for all studied nano-cages.

### H_2_ and CO_2_ Activation

**Figure 2:**
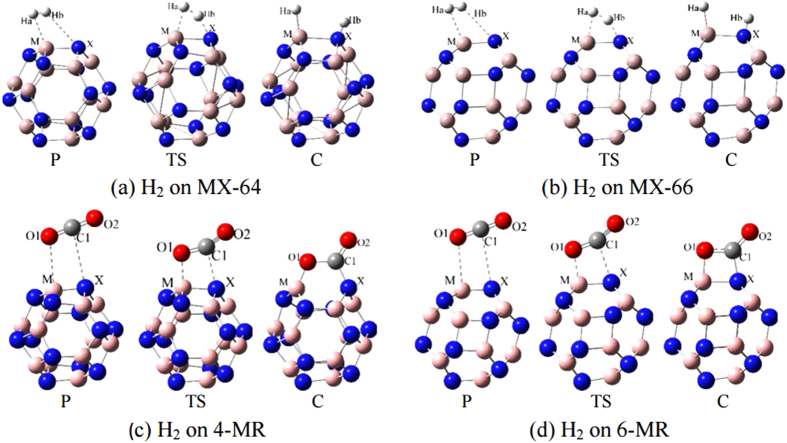
Geometry sketches for H2 and CO2 physiortioon (P), chemisorption (C) and the transition states (TS) in MX-64 and MX-66 configurations.

In order to determine the configuration with the lowest energy for H_2_ and CO_2_ adsorption on the surface of the cluster, a number of different initial structures have been used for optimization. The results of stable H_2_/M_12_x_12_, CO_2_/M_12_x_12_ complexes as well as their corresponding transition state (TS) structures are shown in Fig. 2. For the convenience of discussions, we distinguish the physisorption (P) from the chemical functionalization (C) for the molecules on the cages. As seen from Fig. 2, in the process of physisorption, H_2_ and CO_2_ molecules are weakly adsorbed on the clusters with minor changes in geometry. While in the chemical adsorption, H_2_ is dissociated forming 2H* and CO_2_ is chemically activated forming CO_2_*. The corresponding geometry parameters as well as their TSs are shown in Tables S2 and S3, and the interaction energies of H_2_ and CO_2_ physisorption and chemical absorption are given in Table S4 of Supporting Information.

The activation energies of H_2_ on MX-64 and MX-66 are labeled as 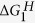 and 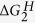, respectively, while the activation energies of CO_2_ on MX-64 and MX-66 are labeled as 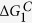 and 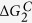 respectively. The energy barriers for H_2_ activation (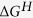) are calculated as the Gibbs energy difference between the 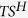 (the TS for H_2_ activation) and the initial state of H_2_ adsorption:


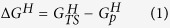


Similarly, the energy barriers for the CO_2_ activation (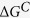) are calculated as the Gibbs energy difference between the 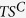 (the TS for CO_2_ activation) and the initial state of CO_2_ adsorption:


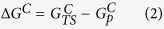


The calculated results are listed in Table 1, and typical structures with H_2_ and CO_2_ either in physisorption, or chemisorption as well as their transition states are given in the Fig. 2. All the Gibbs energy barrier for H_2_ activation on MX-64 are all lower than which of the MX-66. When the activation barrier is overcome, H_2_ can be dissociated generating hydridic (Ha) and protic (Hb) hydrogens.

Instead for CO_2_ activation, the Gibbs energy barrier on MX-64 are all higher than that of the MX-66 except for Al_12_P_12_ and Be_12_O_12_ clusters. The activation barriers of CO_2_ are lower than that of H_2_ for the studied systems except for B_12_P_12_. The Al, Ga doped and Li, Na decorated B_12_N_12_ cages have lower activation energy barriers for H_2_ and CO_2_ than those of the pristine B_12_N_12_. This illustrates that the doping with Al and Ga as well as decoration with Li and Na can increase the activity of B_12_N_12_ cluster.

To clarify the effect of H_2_ and CO_2_ adsorptions on the electronic structures of nano-cages, natural bond orbital (NBO) analyses are performed and the results are listed in Table S5, from which one can see that upon the adsorption, charges on the cages are redistributed due to the geometry change and charge transfer. For example, in all the cases CO_2_ received electrons from cages resulting in the activation. The charges on M sites in all clusters are decreased upon the H_2_ adsorption, while increased upon the CO_2_ adsorption.

The different behaviors in H_2_ activation on MX-64 and MX-66 are due to the different activities between them. As seen from Table S5, more charges are on M and X sites in MX-64 than those in MX-66, which makes the former more active with a lower H_2_ activation barrier as compared with the latter one.

### 2H* transfer mechanism

Two reaction pathways for CO_2_ hydrogenation on Lewis pair moiety have been identified, one involves the physisorped CO_2_ reacting with the chemisorbed 2H*, and the other one involves the physisorped H_2_ reacting with CO_2_*. For the latter, the reaction barrier for hydrogenation of the activated CO_2_* is usually very high as found by Ye^12^ (2.65 eV in UiO-66-P-BF_2_ catalyst) and by us (2.84 eV for MX-64 and 2.97 eV for MX-66 of B_12_N_12_), and this pathway leads to the formation of chemisorbed HCO and OH ([HCO + OH]*) instead of HCOOH as shown in Supporting Information (Fig. S1). Consequently, in the following discussions, we only focused on the first path way for HCOOH formation.

**Figure 3:**
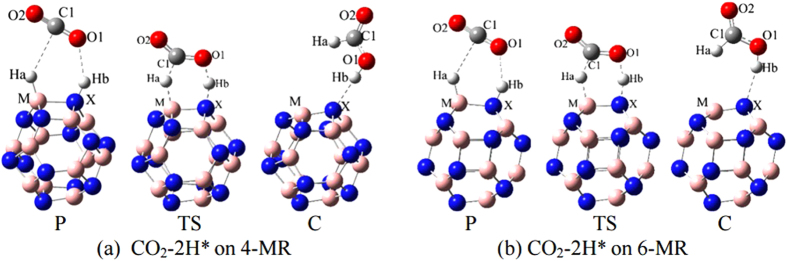
Geometry sketches for 2H* transfer from MX cluster to CO_2_ in physisorption (P), chemisorption (C) and the transition states (TS) on MX-64 (**a**) and MX-66 (**b**) of the MX-2H* clusters.

According to the first pathway, CO_2_ is firstly physiosorbed on MX-2H*(P), and then forms HCOOH (C) via the transition state (TS) (Fig. 3). For B_12_N_12_ nano cage, the reaction barrier for H_2_ activation on MX-64 (1.19 eV) is lower than that on MX-66 (1.27 eV), while the 2H* transfer barrier on MX-64 (1.28 eV) is higher than that on MX-66 (1.19 eV). The IRC calculations show that the hydride and protons are transferred to CO_2_ simultaneously. In other words, the H transfer step is essentially the donation of a hydride and a proton from the ion-pair products to CO_2_ via a concerted mechanism. Tables S6 and S7 list the corresponding geometry parameters, transition states, and interaction energies for CO_2_ and HCOOH.

**Figure 4:**
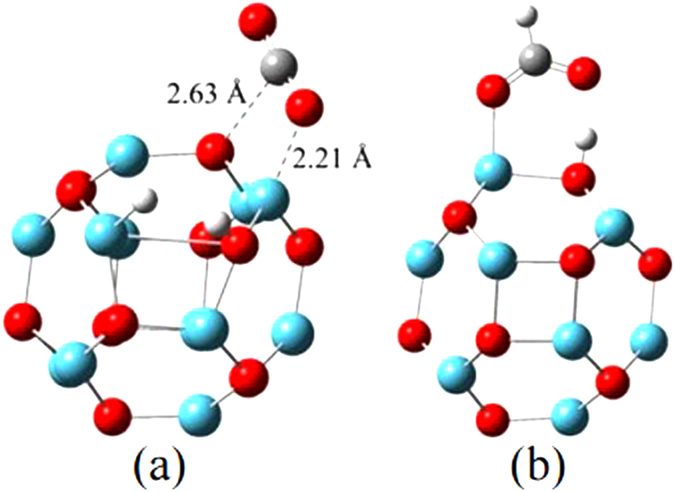
(**a**) Physisorption of CO_2_ on Mg_12_O_12_−2H*; (**b**) HCOOH on Mg_12_O_12_−2H*. (The jade-blue sphere representing Mg atom, red sphere representing O atom, gray sphere representing C atom and white sphere representing H, respectively).

When attempting to bind CO_2_ with Mg_12_O_12_−2H* in a way shown in Fig. 3(P), we cannot find any stable structures from our calculations. While CO_2_ can directly bonds at Mg and O sites (as shown in Fig. 4a) with a stronger binding energy of −0.5 eV (Table S4). Similarly, when introducing HCOOH to Mg_12_O_12_ shown in Fig. 3(C), one H atom of HCOOH was taken away by the O atom in Mg_12_O_12_ nano-cage (Fig. 4b). This suggests that Mg_12_O_12_ is not competent to be the catalyst for CO_2_ hydrogenation to HCOOH.

The activation energies of H_2_ transfer on MX-64 and MX-66 are labeled as 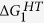 and 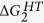 and respectively. The energy barriers for H_2_ activation (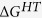) are calculated as the Gibbs energy difference between the TS^HT^ (the TS for H_2_ transfer) and the initial state of CO_2_ the physisorbed on MX-2H*:


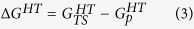


The corresponding activation energies for H_2_ transfer are listed in Table 2, where one can see that the activation energies of H transfer are all higher than 1.0 eV, except for Be_12_O_12_ cage. Just the opposite to the H_2_ activation process in Table 1, all the Gibbs energy barriers for 2H* transfer on MX-64 are all higher than that of MX-66.

Based on the overall consideration of H_2_ activation and 2 H* transfer barriers as listed in Tables 1 and 2, one can find that introducing Al, Ga, Li, and Na to B_12_N_12_ cage has definitely decreased the H_2_ activation energy barrier but increased the 2H transfer energy as compared to the pristine B_12_N_12_.

**Figure 5:**
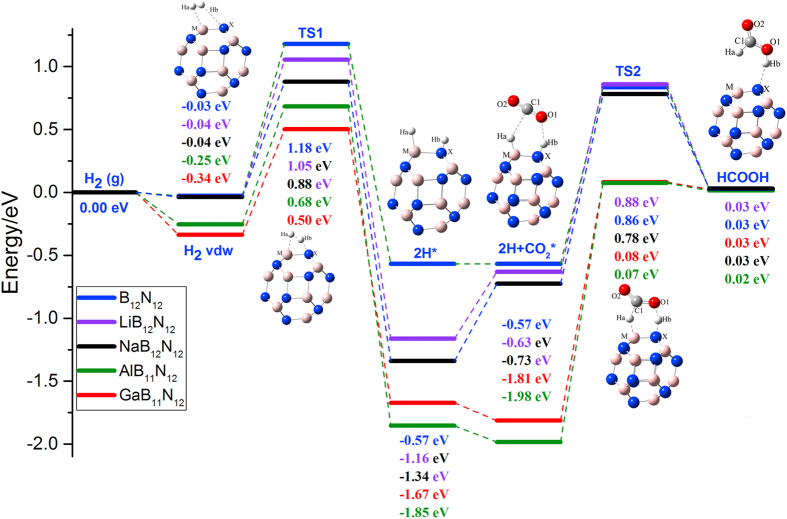
Relative potential energy surfaces for H_2_ activation and 2H* transfer pathway in Al, Ga doped, Li, Na decorated B_12_N_12_ and pristine B_12_N_12_ with Mx-66 bond as example.

In order to be more intuitive, a potential energy surfaces of the reaction pathway are shown in Fig. 5 showing a balance between H_2_ activation and H transfer. Thus, the interaction between the cluster and H_2_ is extremely important, the stronger catalyst with more strength to activate the hydrogen molecule would promote a faster hydrogen activation process. On the other hand, a stronger catalyst has more strength to keep the hydrogen, thus would slow down the hydrogen transfer process. This is in accordance with the general Sabatier principle^43^.

To understand the trend of protonation activation barriers for the studied nanocages, we analyze the charges on C site of CO_2._ In the free standing state, C carries 1.069 e. When CO_2_ is adsorbed with MX-66 configuration on B_12_N_12_-2H*, NaB_12_N_12_-2H* and LiB_12_N_12_-2H*, the charges increase to 1.086, 1.084, and 1.087 e, respectively. The increased charges on C site make it more active to easily bind H with smaller barriers. The similar mechanism can also be applied to other cages.

### 2H* transfer one by one with stepwise mechanism

**Figure 6:**
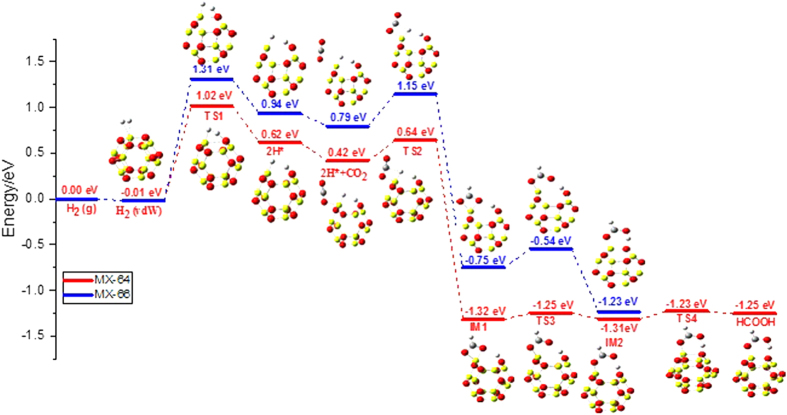
Relative potential energy surfaces for H_2_ activation and H* stepwise transfer pathway on Mx-64 (redline) and MX-66 (blue line) bond for Be_12_O_12_. (The yellow sphere representing Be atom, red sphere representing O atom, gray sphere representing C atom and white sphere representing H, respectively).

When checking the overall the activation energy barrier of H_2_ activation and transfer (seen from Tables 1 and 2), one can find that many clusters such as Al_12_N_12_, NaB_12_N_12_ and AlB_11_N_12_ etc. have lower H_2_ activation but higher 2 H* transfer barriers. To search for a lower barrier of H transfer, the other mechanism needs to be investigated further. We find that a new stepwise mechanism exists for CO_2_ hydrogenation only on Be_12_O_12_ cage as shown in Fig. 6.

By following this reaction mechanism, the first H transfer is a rate-limiting step-with an activation energy of 0.22 eV on MX-64 bond (red line on Fig. 6) and 0.36 eV on the MX-66 bond (blue line on Fig. 6), In contrast, the 2H* transfer activation on MX-64 and MX-66 bonds is 0.45 eV and 0.24 eV, respectively. Then, we can conclude that 2H* simultaneously transfer to CO_2_ on MX-66 bond has lower activation energy than that of the one H transfer stepwise, but on MX-64 bond the situation is opposite.

For practical applications of an efficient catalyst, both the H_2_ activation and transfer barriers should be comparable or lower than 1 eV^44^. Among all systems studied here, Be_12_O_12_ is the most promising catalyst, where the H_2_ activation barrier is close to 1 eV (1.04 eV) on MX-64 bond, and the following H* transfer barriers are all lower than 1 eV, (0.45 eV by the 2H* simultaneously transfer mechanism, while 0.22 eV by the H* stepwise transfer mechanism). Therefore, the reaction pathway on Be_12_O_12_ MX-64 bond has the lowest barrier based on the overall consideration of the H_2_ activation (1.04 eV) and H* stepwise transfer (0.22 eV).

## Conclusions

In summary, based on the DFT and MP2 calculations, a series M_12_X_12_ nano-cages have been studied for activating H_2_ and CO_2_ to form HCOOH. The hydrogenation process mainly consists of H_2_ activation to 2H*, and then 2H* further transfer to CO_2_ forming a HCOOH molecule. Two kinds of H* transfer mechanisms are found: one involves 2H* simultaneous transfer, and the other is a stepwise H* transfer to CO_2_. The two mechanisms result in the same product HCOOH. Moreover, Al, Ga doped and Li, Na decorated B_12_N_12_ cages have lower H_2_ activation energy barriers, but higher 2H* transfer activation barriers than that of the pristine B_12_N_12_. For practical applications, in order to have an efficient catalyst to reduce CO_2_, we should search for a catalyst that has a balance between the energy barriers for H_2_ activation and the H transferring. Among all the systems studied here, Be_12_O_12_ is found to be the most promising catalyst, its reaction pathway on MX-64 bond has the lowest barriers (1.04 eV for H_2_ activation and 0.22 eV for H* transferring). This conclusion would motivate experimental work in the future.

## Methods

Since many theoretical calculations have demonstrated that different DFT functions (e.g. B97D, ω-B97X-D, and M06-2X) and basis sets (e.g. 6–31 G*, 6–31 + G**) led to very similar results for the systems only containing main group elements for H_2_ activations^7^,^8^,^11^. In this work, all the geometry optimizations are performed at the M06-2X/6–31 + G** level as implemented in Gaussian 09 package^45^. Solvent effects are taken into account by using the polarizable continuum model (PCM) with toluene as a solvent. The highly parameterized, empirical exchange correlation functional, M06-2X, developed by Zhao and Truhlar, was shown to better describe the main-group thermochemistry and kinetics than other density functionals such as B3LYP^46^. Moreover, this hybrid density M06-2X functional has been previously proved to have a good reliability in computing molecular binding energies of H_2_ and CO_2_ on FLPs^47^. Frequency calculations are carried out at the same level to characterize the nature of the stationary points along the reaction coordinates. No imaginary frequencies were found for the local minima, and one and only one imaginary frequency was found for the transition state. The Natural Bond Orbital (NBO 3.1) program^48^, was used to calculate the natural charges at the M06-2X/6–31 + G** level of theory. The thermal contributions at room temperature (298.15 K) including the specific free energies were obtained from a harmonic analysis, and accurate electronic energies were obtained from frequency calculations using Møller-Plesset second-order perturbation theory (MP2)^49^,^50^ with the cc-pVTZ triple-ζ quality basis^51^,^52^. Using the optimized geometries and starting from the TS, intrinsic reaction coordinate (IRC) calculations are performed to verify the true connection of the reactants, the transition states and the products for both H_2_, CO_2_ activation and H transfer processes.

## Additional Information

**How to cite this article**: Zhu, H. *et al*. Be_12_O_12_ Nano-cage as a Promising Catalyst for CO_2_ Hydrogenation. *Sci. Rep.* **7**, 40562; doi: 10.1038/srep40562 (2017).

**Publisher's note:** Springer Nature remains neutral with regard to jurisdictional claims in published maps and institutional affiliations.

## Supplementary Material

Supplementary Information

## Figures and Tables

**Table 1:** Gibbs energy barrier for H_2_ (ΔG^H^), and CO_2_ activation (ΔG^C^), All values are in eV.

Clusters	B_12_N_12_	Al_12_N_12_	B_12_P_12_	Al_12_P_12_	Be_12_O_12_	Mg_12_O_12_	LiB_12_N_12_	NaB_12_N_12_	AlB_11_N_12_	GaB_11_N_12_
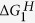	1.19	0.61	0.74	1.25	1.04	0.31	0.99	0.90	0.76	0.83
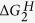	1.27	0.68	1.06	1.46	1.33	0.73	1.09	0.92	0.84	0.94
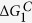	0.56	0.12	1.11	0.61	0.44	0.04	0.56	0.50	0.39	0.46
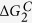	0.51	0.11	1.06	0.63	0.47	0.03	0.33	0.26	0.36	0.43

**Table 2:** Gibbs energy barrier for H_2_ transfer (ΔG^HT^) to form HCOOH, all values are in eV.

Clusters	B_12_N_12_	Al_12_N_12_	B_12_P_12_	Al_12_P_12_	Be_12_O_12_	LiB_12_N_12_	NaB_12_N_12_	AlB_11_N_12_	GaB_11_N_12_
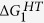	1.45	1.43	2.02	1.41	0.45	2.16	1.75	2.05	2.25
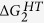	1.40	1.36	1.91	1.47	0.24	1.49	1.51	1.89	2.06
