# Supplementary material for: Be12O12 Nano-cage as a Promising Catalyst for CO2 Hydrogenation
Source: Sci Rep. 2017 Jan 18;7:40562. doi: 10.1038/srep40562 (PMC5241807; doi:10.1038/srep40562)
Supplement: Supplementary Information [file srep40562-s1.docx]

**Be_12_O_12_ Nano-cage as a Promising Catalyst for** **CO_2_** **Hydrogenation**

Haiyan Zhu^1,2^, Yawei Li^1, 3^, Guizhi Zhu^1, 3^, Haibin Su^1, 4^, Siew Hwa Chan^1, 5^ & Qiang Sun^1, 3,^*

^1^ Singapore-Peking University Research Centre, Campus for Research Excellence & Technological Enterprise (CREATE), Singapore 138602

^2^ Institute of Modern Physics, Northwest University, Shaanxi Key Laboratory for Theoretical Physics Frontiers, Xi’an, China 710069

^3^ Department of Materials Science and Engineering, Peking University, Beijing, China 100871

^4^ School of Materials Science and Engineering, Nanyang Technological University, Singapore 639798

^5^ School of Mechanical and Aerospace Engineering, Nanyang Technological University, Singapore 639798

*Corresponding author.

Email: [zhuhaiyan@nwu.edu.cn](mailto:zhuhaiyan@nwu.edu.cn) (Haiyan Zhu)

| **Table S1.** Bond distance (Å) of the clusters. | | | | | | | | | | |
| --- | --- | --- | --- | --- | --- | --- | --- | --- | --- | --- |
| Clusters | B_12_N_12_ | Al_12_N_12_ | B_12_P_12_ | Al_12_P_12_ | Be_12_O_12_ | Mg_12_O_12_ | AlB_11_N_12_ | GaB_11_N_12_ | LiB_12_N_12_ | NaB_12_N_12_ |
| B(M1,X1) | 1.48 | 1.85 | 1.92 | 2.33 | 1.58 | 1.94 | 1.50 | 1.50 | 1.51 | 1.52 |
| B(M1,X2) | 1.44 | 1.79 | 1.91 | 2.28 | 1.53 | 1.89 | 1.43 | 1.43 | 1.45 | 1.47 |
| B(M2,X2) |  |  |  |  |  |  | 1.83 | 1.90 |  |  |
| B(M2,X3) |  |  |  |  |  |  | 1.79 | 1.85 |  |  |
| B(M3,X1) |  |  |  |  |  |  |  |  | 2.34 | 2.39 |
| B(M3,M1) |  |  |  |  |  |  |  |  | 2.34 | 2.22 |

| **Table S2.** Bond length (Å) for H_2_ physiortioon (P), chemisorption (C) and the transition states (TS) on the clusters. | | | | | | | | | |
| --- | --- | --- | --- | --- | --- | --- | --- | --- | --- |
| Clusters | | H_2_ on MX-64 | | | | H_2_ on MX-66 | | | |
|  |  | B(M,Ha) | B(X,Hb) | B(Ha,Hb) | B(M,X) | B(M,Ha) | B(X,Hb) | B(Ha,Hb) | B(M,X) |
| B_12_N_12_ | P | 2.78 | 3.92 | 0.74 | 1.48 | 3.30 | 2.93 | 0.74 | 1.44 |
|  | TS | 1.34 | 1.41 | 0.99 | 1.69 | 1.33 | 1.37 | 1.02 | 1.59 |
|  | C | 1.20 | 1.02 | 2.56 | 1.74 | 1.20 | 1.02 | 2.38 | 1.66 |
| Al_12_N_12_ | P | 2.09 | 3.00 | 0.75 | 1.86 | 2.13 | 2.76 | 0.75 | 1.79 |
|  | TS | 1.79 | 1.44 | 0.97 | 1.97 | 1.80 | 1.45 | 0.97 | 1.88 |
|  | C | 1.59 | 1.02 | 2.88 | 2.12 | 1.59 | 1.02 | 2.66 | 2.00 |
| B_12_P_12_ | P | 3.47 | 3.38 | 0.74 | 1.92 | 2.98 | 3.96 | 0.74 | 1.91 |
|  | TS | 1.27 | 1.85 | 1.06 | 2.16 | 1.24 | 1.63 | 1.24 | 2.20 |
|  | C | 1.19 | 1.41 | 3.23 | 2.00 | 1.20 | 1.41 | 2.98 | 1.96 |
| Al_12_P_12_ | P | 2.89 | 3.99 | 0.74 | 2.33 | 3.17 | 3.40 | 0.74 | 2.28 |
|  | TS | 1.69 | 1.70 | 1.12 | 2.61 | 1.68 | 1.63 | 1.21 | 2.49 |
|  | C | 1.58 | 1.41 | 3.84 | 2.50 | 1.58 | 1.41 | 3.64 | 2.43 |
| LiB_12_N_12_ | P | 2.74 | 3.15 | 0.74 | 1.49 | 2.72 | 3.08 | 0.74 | 1.44 |
|  | TS | 1.33 | 1.42 | 0.99 | 1.71 | 1.33 | 1.38 | 1.01 | 1.60 |
|  | C | 1.19 | 1.02 | 2.49 | 1.71 | 1.19 | 1.02 | 2.32 | 1.65 |
| NaB_12_N_12_ | P | 2.72 | 3.03 | 0.74 | 1.50 | 2.74 | 3.01 | 0.74 | 1.45 |
|  | TS | 1.33 | 1.43 | 0.98 | 1.73 | 1.35 | 1.45 | 0.98 | 1.56 |
|  | C | 1.19 | 1.02 | 2.53 | 1.79 | 1.20 | 1.02 | 2.32 | 1.65 |
| AlB_11_N_12_ | P | 2.00 | 2.93 | 0.75 | 1.84 | 1.98 | 2.88 | 0.75 | 1.80 |
|  | TS | 1.74 | 1.46 | 0.98 | 1.95 | 1.74 | 1.44 | 0.99 | 1.89 |
|  | C | 1.56 | 1.02 | 3.32 | 2.05 | 1.57 | 1.02 | 3.01 | 1.98 |
| GaB_11_N_12_ | P | 2.11 | 3.10 | 0.76 | 1.90 | 2.09 | 3.15 | 0.76 | 1.86 |
|  | TS | 1.77 | 1.52 | 0.96 | 2.02 | 1.76 | 1.49 | 0.98 | 1.95 |
|  | C | 1.56 | 1.02 | 3.40 | 2.09 | 1.56 | 1.02 | 3.08 | 2.02 |
| Be_12_O_12_ | P | 2.04 | 2.65 | 0.74 | 1.59 | 2.04 | 2.60 | 0.74 | 1.53 |
|  | TS | 1.51 | 1.29 | 0.96 | 2.22 | 1.50 | 1.14 | 1.13 | 1.81 |
|  | C | 1.40 | 0.98 | 1.81 | 2.67 | 1.38 | 0.98 | 1.62 | 2.81 |
| Mg_12_O_12_ | P | 2.44 | 2.53 | 0.75 | 1.94 | 2.44 | 2.96 | 0.74 | 1.89 |
|  | TS | 2.04 | 1.26 | 1.00 | 2.10 | 1.95 | 1.23 | 1.03 | 2.03 |
|  | C | 1.80 | 0.97 | 1.88 | 2.95 | 1.81 | 0.97 | 2.25 | 2.13 |

| **Table S3.** Geometry parameters for CO_2_ physisorptioon (P), chemisorption (C) and the transition states (TS) on the clusters. Bond length B (Å). Bond angle A (^O^) | | | | | | | | | | | |
| --- | --- | --- | --- | --- | --- | --- | --- | --- | --- | --- | --- |
| Clusters | | CO_2_ on MX-64 | | | | | CO_2_ on MX-66 | | | | |
|  |  | B(M,O1) | B(X,C1) | B(O1,C1) | B(M,X) | A(O1,C1,O2) | B(M,O1) | B(X,C1) | B(O1,C1) | B(M,X) | A(O1,C1,O2) |
| B_12_N_12_ | P | 2.62 | 2.99 | 1.17 | 1.49 | 179.11 | 2.58 | 2.99 | 1.17 | 1.45 | 179.48 |
|  | TS | 1.63 | 2.06 | 1.23 | 1.59 | 156.82 | 1.64 | 2.07 | 1.22 | 1.53 | 157.85 |
|  | C | 1.40 | 1.43 | 1.35 | 2.52 | 123.00 | 1.48 | 1.48 | 1.32 | 1.67 | 132.20 |
| Al_12_N_12_ | P | 2.06 | 2.83 | 1.18 | 1.89 | 175.88 | 2.06 | 2.82 | 1.18 | 1.82 | 175.89 |
|  | TS | 1.99 | 2.33 | 1.20 | 1.91 | 161.96 | 1.99 | 2.34 | 1.20 | 1.84 | 162.65 |
|  | C | 1.90 | 1.35 | 1.30 | 2.14 | 127.03 | 1.81 | 1.45 | 1.33 | 2.00 | 126.68 |
| B_12_P_12_ | P | 3.02 | 3.47 | 1.16 | 1.92 | 179.77 | 2.99 | 3.50 | 1.16 | 1.91 | 179.85 |
|  | TS | 1.63 | 2.39 | 1.23 | 2.00 | 151.37 | 1.64 | 2.41 | 1.23 | 1.97 | 152.37 |
|  | C | 1.37 | 1.88 | 1.37 | 3.31 | 119.51 | 1.52 | 1.94 | 1.31 | 1.99 | 130.95 |
| Al_12_P_12_ | P | 2.22 | 3.46 | 1.18 | 2.35 | 178.76 | 2.22 | 3.43 | 1.18 | 2.31 | 178.68 |
|  | TS | 1.96 | 2.46 | 1.23 | 2.42 | 151.72 | 1.95 | 2.43 | 1.23 | 2.35 | 150.11 |
|  | C | 1.74 | 1.90 | 1.33 | 3.72 | 122.18 | 1.84 | 1.95 | 1.31 | 2.41 | 129.31 |
| LiB_12_N_12_ | P | 2.56 | 3.05 | 1.17 | 1.50 | 179.64 | 2.52 | 3.06 | 1.17 | 1.45 | 179.72 |
|  | TS | 1.61 | 2.05 | 1.23 | 1.61 | 156.11 | 1.64 | 2.21 | 1.22 | 1.50 | 162.91 |
|  | C | 1.38 | 1.41 | 1.37 | 2.56 | 120.59 | 1.46 | 1.46 | 1.33 | 1.67 | 130.63 |
| NaB_12_N_12_ | P | 2.57 | 2.97 | 1.17 | 1.51 | 179.47 | 2.52 | 3.05 | 1.17 | 1.47 | 179.79 |
|  | TS | 1.61 | 2.06 | 1.23 | 1.62 | 156.35 | 1.64 | 2.25 | 1.21 | 1.50 | 162.67 |
|  | C | 1.40 | 1.41 | 1.35 | 2.44 | 122.38 | 1.46 | 1.46 | 1.34 | 1.67 | 130.43 |
| AlB_11_N_12_ | P | 1.94 | 3.45 | 1.19 | 1.85 | 178.07 | 1.93 | 3.87 | 1.18 | 1.81 | 178.40 |
|  | TS | 1.89 | 2.16 | 1.23 | 1.90 | 156.09 | 1.89 | 2.16 | 1.23 | 1.85 | 157.03 |
|  | C | 1.78 | 1.47 | 1.33 | 2.11 | 127.39 | 1.79 | 1.48 | 1.33 | 1.97 | 129.03 |
| GaB_11_N_12_ | P | 2.07 | 3.50 | 1.18 | 1.91 | 177.40 | 2.10 | 3.15 | 1.19 | 1.86 | 177.32 |
|  | TS | 1.99 | 2.12 | 1.23 | 1.85 | 157.03 | 1.99 | 2.12 | 1.22 | 1.90 | 155.40 |
|  | C | 1.87 | 1.47 | 1.32 | 2.14 | 127.47 | 1.88 | 1.48 | 1.32 | 2.01 | 129.00 |
| Be_12_O_12_ | P | 1.95 | 2.70 | 1.17 | 1.63 | 177.88 | 1.91 | 2.67 | 1.18 | 1.57 | 177.42 |
|  | TS | 1.74 | 1.92 | 1.21 | 1.76 | 154.33 | 1.74 | 1.89 | 1.22 | 1.65 | 153.63 |
|  | C | 1.57 | 1.42 | 1.29 | 2.56 | 128.96 | 1.54 | 1.39 | 1.29 | 2.58 | 127.68 |
| Mg_12_O_12_ | P | 2.24 | 2.54 | 1.18 | 1.97 | 172.33 | 2.24 | 2.56 | 1.18 | 1.91 | 172.81 |
|  | TS | 2.14 | 2.19 | 1.19 | 2.00 | 161.90 | 2.22 | 2.27 | 1.19 | 1.92 | 164.61 |
|  | C | 2.04 | 1.35 | 1.27 | 2.31 | 128.30 | 2.08 | 1.35 | 1.26 | 2.08 | 128.25 |

| **Table S4.** Binding energies for H_2_ physisorption ($E_{P}^{H}$) and chemical functionalization ($E_{C}^{H}$) as well as CO_2_ physisorption ($E_{P}^{C}$) and chemical functionalization ($E_{C}^{C}$) in CPCM model (using toluene as the solvent (S). All values are in eV. | | | | | | | | | | | | |
| --- | --- | --- | --- | --- | --- | --- | --- | --- | --- | --- | --- | --- |
| Clusters | | B_12_N_12_ | Al_12_N_12_ | B_12_P_12_ | Al_12_P_12_ | Be_12_O_12_ | Mg_12_O_12_ | LiB_12_N_12_ | NaB_12_N_12_ | AlB_11_N_12_ | GaB_11_N_12_ |  |
| H_2_ on  MX-64 | P | -0.03 | -0.11 | -0.03 | -0.03 | -0.01 | 0.19 | -0.03 | -0.03 | -0.32 | -0.25 |  |
|  | C | -0.59 | -1.16 | -0.32 | 0.41 | 0.62 | 0.15 | -1.17 | -0.45 | -1.80 | -1.99 |  |
| H_2_ on  MX-66 | P | -0.03 | -0.11 | -0.03 | 0.62 | -0.01 | 0.01 | -0.04 | -0.04 | -0.34 | -0.25 |  |
|  | C | -0.57 | -1.20 | -0.15 | 0.62 | 0.94 | 0.15 | -1.16 | -1.18 | -1.67 | -1.85 |  |
| CO_2_ on  MX-64 | P | -0.20 | -0.55 | -0.14 | -0.30 | -0.34 | -0.33 | -0.22 | -0.23 | -0.96 | -0.69 |  |
|  | C | -0.20 | -0.55 | -0.14 | -0.30 | -0.34 | -0.33 | -0.22 | -0.23 | -0.96 | -0.69 |  |
| CO_2_ on  MX-66 | P | -0.20 | -0.54 | -0.13 | -0.29 | -0.33 | -0.32 | -0.22 | -0.22 | -0.957352019 | -0.69 |  |
|  | C | 0.05 | -1.50 | 0.99 | 0.53 | 0.26 | -1.48 | -0.61 | -0.80 | -0.90 | -0.53 |  |

| **Table S5.** NBO charge population for different pristine as well as H_2_ and CO_2_ chemical adsorbed nano cages. | | | | | | | | | | | |
| --- | --- | --- | --- | --- | --- | --- | --- | --- | --- | --- | --- |
| Clusters | | B_12_N_12_ | Al_12_N_12_ | B_12_P_12_ | Al_12_P_12_ | Be_12_O_12_ | Mg_12_O_12_ | LiB_12_N_12_ | NaB_12_N_12_ | AlB_11_N_12_ | GaB_11_N_12_ |
| Pristine  Cluster | M_0_ | 1.19 | 2.00 | -0.23 | 1.04 | 1.71 | 1.84 | 1.09 | 1.17 | 2.11 | 1.97 |
|  | X_0_ | -1.19 | -2.00 | 0.23 | -1.04 | -1.71 | -1.84 | -1.16 | -1.20 | -1.39 | -1.33 |
| H_2_ on  MX-64 | M | 0.74 | 1.72 | -0.60 | 1.00 | 1.54 | 1.66 | 0.71 | 0.74 | 1.74 | 1.61 |
|  | X | -1.20 | -1.80 | 0.44 | -0.82 | -1.40 | -1.46 | -1.22 | -1.22 | -1.34 | -1.32 |
|  | Ha | -0.05 | -0.43 | 0.05 | -0.34 | -0.67 | -0.73 | -0.04 | -0.05 | -0.38 | -0.32 |
|  | Hb | 0.51 | 0.47 | 0.11 | 0.13 | 0.54 | 0.52 | 0.51 | 0.52 | 0.50 | 0.50 |
| H_2_ on  MX-66 | M | 0.71 | 1.70 | -0.65 | 0.96 | 1.51 | 1.64 | 0.68 | 0.69 | 1.73 | 1.60 |
|  | X | -1.17 | -1.79 | 0.46 | -0.78 | -1.42 | -1.46 | -1.21 | -1.20 | -1.33 | -1.30 |
|  | Ha | -0.05 | -0.42 | 0.05 | -0.34 | -0.63 | -0.75 | -0.04 | -0.04 | -0.38 | -0.32 |
|  | Hb | 0.51 | 0.47 | 0.11 | 0.12 | 0.53 | 0.54 | 0.51 | 0.51 | 0.50 | 0.50 |
| CO_2_ on  MX-64 | M | 1.36 | 2.05 | 0.45 | 1.50 | 1.72 | 1.83 | 1.35 | 1.34 | 2.13 | 2.02 |
|  | X | -1.05 | -1.45 | 0.40 | -0.45 | -1.19 | -1.10 | -1.07 | -1.07 | -1.19 | -1.20 |
|  | O1 | -0.74 | -0.81 | -0.78 | -0.98 | -0.95 | -0.79 | -0.76 | -0.75 | -0.87 | -0.84 |
|  | C1 | 0.99 | 0.92 | 0.60 | 0.64 | 1.05 | 0.98 | 1.00 | 1.00 | 0.98 | 0.98 |
|  | O2 | -0.56 | -0.78 | -0.55 | -0.60 | -0.66 | -0.79 | -0.56 | -0.58 | -0.58 | -0.58 |
| CO_2_ on  MX-66 | M | 1.20 | 2.01 | -0.09 | 1.28 | 1.70 | 1.82 | 1.17 | 1.17 | 2.10 | 1.99 |
|  | X | -1.10 | -1.60 | 0.45 | -0.78 | -1.21 | -1.13 | -1.11 | -1.11 | -1.23 | -1.21 |
|  | O1 | -0.69 | -0.87 | -0.68 | -0.86 | -0.96 | -0.86 | -0.70 | -0.71 | -0.85 | -0.82 |
|  | C1 | 1.03 | 0.96 | 0.72 | 0.73 | 1.07 | 1.02 | 1.02 | 1.02 | 0.99 | 0.99 |
|  | O2 | -0.56 | -0.65 | -0.55 | -0.58 | -0.66 | -0.87 | -0.56 | -0.57 | -0.57 | -0.58 |

**Fig. S1.** Potential energy surfaces for two different CO_2_ hydrogenation pathways on MX-66 and MX-64 of B_12_N_12._

| **Table. S6.** Geometry parameters for CO_2_ physisorptioon(P), chemisorption (C) and the transition states (TS) on the clusters with 2H*. Bond length (Å). Bond angle (^O^) | | | | | | | | |
| --- | --- | --- | --- | --- | --- | --- | --- | --- |
| Clusters | | CO_2_ on MX-64 | | | | | | |
|  |  | B(M,Ha) | B(X,Hb) | B(C1,Ha) | B(O1,Hb) | B(C1,O1) | B(M,X) | A(O1,C1,O2) |
| B_12_N_12_ | P | 1.20 | 1.02 | 2.72 | 2.13 | 1.17 | 1.73 | 179.16 |
|  | TS | 1.46 | 1.16 | 1.26 | 1.35 | 1.25 | 1.60 | 138.63 |
|  | HCOOH | 3.75 | 1.93 | 1.10 | 0.98 | 1.33 | 1.50 | 122.66 |
| Al_12_N_12_ | P | 1.60 | 1.02 | 2.84 | 3.04 | 1.17 | 2.10 | 178.32 |
|  | TS | 2.14 | 1.26 | 1.16 | 1.23 | 1.28 | 1.92 | 130.57 |
|  | HCOOH | 3.54 | 1.65 | 1.11 | 1.02 | 1.32 | 1.88 | 123.84 |
| B_12_P_12_ | P | 1.20 | 1.41 | 3.13 | 3.11 | 1.17 | 1.99 | 179.69 |
|  | TS | 1.37 | 1.57 | 1.35 | 1.37 | 1.24 | 1.96 | 142.32 |
|  | HCOOH | 3.25 | 2.59 | 1.10 | 0.97 | 1.34 | 1.92 | 122.29 |
| Al_12_P_12_ | P | 1.58 | 1.41 | 3.22 | 2.81 | 1.17 | 2.49 | 179.41 |
|  | TS | 1.81 | 1.47 | 1.26 | 1.65 | 1.23 | 2.41 | 138.85 |
|  | HCOOH | 3.57 | 2.50 | 1.10 | 0.97 | 1.34 | 2.33 | 122.45 |
| LiB_12_N_12_ | P | 1.20 | 1.02 | 2.77 | 2.62 | 1.17 | 1.71 | 179.24 |
|  | TS | 1.46 | 1.20 | 1.27 | 1.30 | 1.25 | 1.59 | 140.61 |
|  | HCOOH | 2.93 | 2.00 | 1.10 | 0.98 | 1.34 | 1.62 | 122.16 |
| NaB_12_N_12_ | P | 1.20 | 1.02 | 2.71 | 2.15 | 1.17 | 1.74 | 179.46 |
|  | TS | 1.46 | 1.21 | 1.26 | 1.28 | 1.25 | 1.60 | 138.39 |
|  | HCOOH | 2.92 | 1.99 | 1.10 | 0.98 | 1.33 | 1.63 | 122.18 |
| AlB_11_N_12_ | P | 1.57 | 1.02 | 2.88 | 2.48 | 1.17 | 2.04 | 179.63 |
|  | TS | 1.83 | 1.30 | 1.24 | 1.88 | 1.26 | 1.91 | 135.77 |
|  | HCOOH | 3.91 | 1.86 | 1.10 | 0.98 | 1.33 | 1.85 | 123.01 |
| GaB_11_N_12_ | P | 1.56 | 1.02 | 2.96 | 2.71 | 1.17 | 2.08 | 179.32 |
|  | TS | 1.82 | 1.32 | 1.23 | 1.17 | 1.27 | 1.95 | 135.03 |
|  | HCOOH | 2.05 | 1.66 | 1.15 | 1.02 | 1.30 | 1.92 | 128.20 |
| Be_12_O_12_ | P | 1.39 | 0.97 | 2.58 | 2.37 | 1.17 | 2.69 | 177.34 |
|  | TS | 1.42 | 0.97 | 1.71 | 1.86 | 1.19 | 2.75 | 155.43 |
|  | HCOOH | 3.12 | 1.67 | 1.10 | 0.99 | 1.33 | 1.61 | 123.34 |
| CO_2_ on MX-66 | | | | | | | | |
| B_12_N_12_ | P | 1.20 | 1.02 | 2.70 | 2.12 | 1.17 | 1.65 | 179.21 |
|  | TS | 1.46 | 1.17 | 1.26 | 1.34 | 1.25 | 1.54 | 138.57 |
|  | C | 3.22 | 1.94 | 1.10 | 0.98 | 1.33 | 1.45 | 122.82 |
| Al_12_N_12_ | P | 1.60 | 1.02 | 2.62 | 2.19 | 1.17 | 1.99 | 178.12 |
|  | TS | 2.13 | 1.27 | 1.16 | 1.22 | 1.28 | 1.84 | 130.68 |
|  | HCOOH | 3.40 | 1.65 | 1.11 | 1.02 | 1.32 | 1.81 | 124.00 |
| B_12_P_12_ | P | 1.20 | 1.41 | 283 | 3.08 | 1.17 | 1.96 | 179.34 |
|  | TS | 1.38 | 1.57 | 1.32 | 1.38 | 1.24 | 1.94 | 141.29 |
|  | HCOOH | 3.65 | 2.59 | 1.10 | 0.97 | 1.34 | 1.91 | 122.25 |
| Al_12_P_12_ | P | 1.59 | 1.41 | 2.99 | 3.36 | 1.17 | 2.43 | 179.34 |
|  | TS | 1.84 | 1.48 | 1.25 | 1.59 | 1.24 | 2.35 | 137.76 |
|  | HCOOH | 3.41 | 2.63 | 1.10 | 0.97 | 1.34 | 2.28 | 122.25 |
| LiB_12_N_12_ | P | 1.20 | 1.02 | 2.75 | 2.11 | 1.17 | 1.73 | 179.24 |
|  | TS | 1.43 | 1.17 | 1.28 | 1.34 | 1.25 | 1.58 | 139.56 |
|  | HCOOH | 3.65 | 1.96 | 1.10 | 0.98 | 1.33 | 1.48 | 122.58 |
| NaB_12_N_12_ | P | 1.20 | 1.02 | 2.69 | 2.10 | 1.17 | 1.81 | 179.20 |
|  | TS | 1.44 | 1.17 | 1.28 | 1.34 | 1.25 | 1.61 | 139.42 |
|  | HCOOH | 3.80 | 1.95 | 1.10 | 0.98 | 1.33 | 1.50 | 122.49 |
| AlB_11_N_12_ | P | 1.57 | 1.02 | 2.70 | 2.19 | 1.17 | 1.98 | 179.14 |
|  | TS | 1.83 | 1.30 | 1.24 | 1.18 | 1.26 | 1.86 | 135.86 |
|  | HCOOH | 3.90 | 1.86 | 1.10 | 0.98 | 1.33 | 1.85 | 123.01 |
| GaB_11_N_12_ | P | 1.56 | 1.02 | 2.67 | 2.19 | 1.17 | 2.01 | 179.29 |
|  | TS | 1.82 | 1.32 | 1.23 | 1.16 | 1.27 | 1.91 | 135.02 |
|  | HCOOH | 2.08 | 1.65 | 1.15 | 1.02 | 1.30 | 1.87 | 128.12 |
| Be_12_O_12_ | P | 1.40 | 0.97 | 2.51 | 1.92 | 1.17 | 1.86 | 176.21 |
|  | TS | 1.43 | 0.98 | 1.88 | 1.78 | 1.19 | 1.81 | 160.80 |
|  | C | 3.09 | 1.67 | 1.11 | 0.99 | 1.33 | 1.55 | 123.35 |

| **Table.S7.** Binding energies for CO_2_ physisorption ($E_{P}^{C}$) and chemical functionalization ($E_{C}^{C}$) and their transition states ($E_{TS}^{C}$) of the nano-cage with 2H*. All values are in eV. | | | | | | | | | | |
| --- | --- | --- | --- | --- | --- | --- | --- | --- | --- | --- |
| Clusters | | B_12_N_12_ | Al_12_N_12_ | B_12_P_12_ | Al_12_P_12_ | Be_12_O_12_ | LiB_12_N_12_ | NaB_12_N_12_ | AlB_11_N_12_ | GaB_11_N_12_ |
| CO_2_ on  MX-64 | $E_{P}^{C}$ | -0.01 | 0.36 | -0.18 | -0.18 | -0.17 | -0.24 | -0.67 | -0.18 | -0.18 |
|  | $E_{TS}^{C}$ | 1.45 | 1.79 | 1.83 | 1.23 | 0.28 | 1.92 | 1.08 | 1.86 | 2.06 |
|  | $E_{C}^{C}$ | 0.61 |  | 0.48 | -0.27 | -0.77 | 1.20 | 0.41 | 1.76 | 1.99 |
| CO_2_ on  MX-66 | $E_{P}^{C}$ | 0.00 | -1.33 | -0.20 | -0.38 | -0.10 | 0.53 | 0.46 | -0.14 | -0.13 |
|  | $E_{TS}^{C}$ | 1.40 | 0.02 | 1.72 | 1.02 | 0.14 | 2.02 | 1.96 | 1.75 | 1.93 |
|  | $E_{C}^{C}$ | 0.60 | 0.15 | 0.31 | -0.57 | -1.08 | 1.19 | 1.21 | 1.70 | 1.87 |
